# Update on the Genetics of Systemic Lupus Erythematosus: Genome-Wide Association Studies and Beyond

**DOI:** 10.3390/cells8101180

**Published:** 2019-09-30

**Authors:** Young-Chang Kwon, Sehwan Chun, Kwangwoo Kim, Anselm Mak

**Affiliations:** 1 Department of Rheumatology, Hanyang University Hospital for Rheumatic Diseases, 222–1 Wangsimni-ro, Seongdong-gu, Seoul 04763, Korea; yckwon83@hanyang.ac.kr; 2 Department of Biology, Kyung Hee University, 26 Kyungheedae-ro, Dongdaemun-gu, Seoul 02447, Korea; chunsehwan0922@gmail.com; 3 Division of Rheumatology, University Medicine Cluster, National University Health System, Singapore 119228, Singapore; 4 Department of Medicine, Yong Loo Lin School of Medicine, National University of Singapore, Singapore 119228, Singapore

**Keywords:** genetics, epigenetics, genome, lupus, SLE

## Abstract

Systemic lupus erythematosus (SLE) is a chronic autoimmune disease of complex etiology that primarily affects women of childbearing age. The development of SLE is attributed to the breach of immunological tolerance and the interaction between SLE-susceptibility genes and various environmental factors, resulting in the production of pathogenic autoantibodies. Working in concert with the innate and adaptive arms of the immune system, lupus-related autoantibodies mediate immune-complex deposition in various tissues and organs, leading to acute and chronic inflammation and consequent end-organ damage. Over the past two decades or so, the impact of genetic susceptibility on the development of SLE has been well demonstrated in a number of large-scale genetic association studies which have uncovered a large fraction of genetic heritability of SLE by recognizing about a hundred SLE-susceptibility loci. Integration of genetic variant data with various omics data such as transcriptomic and epigenomic data potentially provides a unique opportunity to further understand the roles of SLE risk variants in regulating the molecular phenotypes by various disease-relevant cell types and in shaping the immune systems with high inter-individual variances in disease susceptibility. In this review, the catalogue of SLE susceptibility loci will be updated, and biological signatures implicated by the SLE-risk variants will be critically discussed. It is optimistically hoped that identification of SLE risk variants will enable the prognostic and therapeutic biomarker armamentarium of SLE to be strengthened, a major leap towards precision medicine in the management of the condition.

## 1. Introduction

### 1.1. Pathophysiology of SLE at a Glance

Systemic lupus erythematosus (SLE) is a highly complex and heterogeneous autoimmune condition that mainly affects women in their reproductive years [1,2]. Despite the advent of disease classification, monitoring strategy and therapeutic options, survival of SLE patients has not improved much further since the 1980s [3]. The heterogeneous nature of the disease and the lack of full understanding of the pathogenic mechanism are major hurdles to further improving the current management strategy of SLE. Beyond survival, vocational ability, health-related quality of life, absenteeism and morbidity are also negatively affected in patients with SLE [4]. 

Despite our partial understanding of the clinical and immunological behavior of the disease, the onset of SLE is postulated to be triggered by environmental and hormonal factors in genetically susceptible individuals [5,6]. Clinical observation and epidemiological studies suggest that environmental factors such as ultraviolet (UV) light, various viral and bacterial infections, alcohol consumption, cigarette smoke, vitamin D deficiency and some occupationally and non-occupationally related chemicals are potential triggers of SLE onset and disease flare [6]. Being a female predominant condition, hormonal factors have long been recognized to play a pathogenically crucial role in SLE [7]. High quality data which demonstrated that lupus patients who are pregnant or on hormonal replacement therapy experience more frequent disease flares further strengthen the relationship between female sex hormones and the pathogenesis of SLE [8,9,10]. Even in physiological concentration, estrogens facilitate humoral immune response by stimulating B-cell proliferation and subsequent autoantibody production [11,12]. Furthermore, estrogens stimulate calcineurin expression which enhances expression of the X-linked gene product CD40L on lupus CD4^+^ T cells, resulting in their strengthened crosstalk with CD40-expressing B cells and autoantibody production in SLE patients [13]. Interestingly, the X chromosome per se contributes to SLE susceptibility independently of the effects of the hormones. For instance, transgenic male mice overexpressing the genes on the X chromosome were endowed with an increased susceptibility to develop lupus-like manifestations when they were treated with pristane [14]. Such observations highlight the importance of CD40 in the pathogenesis of SLE at the genetic rather than the protein level since the overexpressed *CD40L* gene is X-linked.

### 1.2. Interaction between Environmental and Genetic Factors in SLE

It is strongly believed that disease-triggering factors interact with genomic and epigenomic mechanisms, which lead to enhancement of pro-inflammatory and/or suppression of anti-inflammatory responses in individuals susceptible to SLE [15]. Briefly, epigenetic mechanisms such as DNA methylation and histone modification that silence the transcription of genes responsible for initiating and perpetuating pro-inflammatory responses are affected in SLE patients, particularly in their CD4^+^ T cells [16]. Partly due to the deficiency and inhibition of DNA methyltransferase 1 (Dnmt1), an enzyme crucial to maintain DNA methylation [17], the DNA of lupus CD4^+^ T cells is generally hypomethylated [6]. Hypomethylation of DNA tilts lupus CD4^+^ T cells towards autoreactivity, facilitating the production of pro-inflammatory chemokines and cytokines, autoantibodies and polyclonal expansion of autoreactive B cells via T-B cell crosstalk [18]. 

### 1.3. The Roles of Germinal Centre and IgA Deficiency in SLE

Apart from autoreactive antibody formation, recent data have suggested that antigen-specific germinal center response and B cell selection are impaired in murine lupus models (TMPD-induced model, Bm12 cGVHD model and SHIP^ΔB^ spontaneous lupus model) and human SLE, leading to compromised antigen-specific antibody affinity maturation and excessive self-reactive antibody responses in lupus germinal centers [19]. In these lupus models and SLE patients, excessive CD11c^+^Tbet^+^ age-associated B cells were shown to induce dysregulated follicular T-helper cell differentiation, disrupting the latter to execute their potent antigen-presenting function and high-affinity selection of B cells, with subsequent paradoxical coexistence of excessive autoreactive antibodies and insufficiently affinity-matured pathogen-specific antibodies in SLE [19]. Interestingly, inhibition of TLR7 signaling ablated MYD88, leading to inhibition of the differentiation of CD11c^+^Tbet^+^ cells, restoration of follicular T-helper cell functions, resumption of antigen-specific B cell selection and inhibition of autoreactive antibody formation [19]. As far as B cells are concerned, aberrant expression of the chemokine receptor CXCR4 on lupus B cells might contribute to subsequent autoantibody production [20]. In healthy situations, down-regulation of CXCR4 expression on centroblasts in the dark zone of the germinal centers where somatic hypermutation takes place is important because upon repatriation of these centroblasts to the light zone of the germinal centers, affinity-driven selection for B cells can take place. Failed downregulation of CXCR4 found in SLE blocks the re-entry of centroblasts to the light zone, leading to impaired B cell selection and release of autoreactive B cells to the circulation [21].

While autoantibodies are abundant in SLE, IgA deficiency has been implicated in the pathogenesis of SLE. The prevalence of IgA deficiency (~2.6–5.2%) is higher in lupus patients compared with that of the general population (ranges from 1 in 400 to 3000) [22,23,24,25]. In a prevalent study of 96 patients performed in Europe, those with IgA deficiency were more likely to be positive for anti-Sm and anti-La antibodies, although the overall clinical picture of these lupus patients is comparable to those with adequate IgA levels [22]. While how exactly IgA deficiency is related to the pathogenesis of SLE requires further investigation, IgA deficiency should be identified in SLE patients especially if blood transfusion or intravenous immunoglobulin (IVIg) therapy is contemplated. Transfusion of blood products devoid of anti-IgA antibodies coupled with meticulous monitoring for transfusion or infusion reaction is necessary in IgA-deficient SLE patients.

### 1.4. Breach of Immune Tolerance in SLE

Working in concert with disease-triggering factors, loss of the central and peripheral immunological tolerance mechanisms has been implicated as a major pathogenic driver of SLE [26]. Peripherally, inefficacious clearance of immune complexes and accumulation of apoptotic cells (carrying nuclear autoantigens) expose the intolerant immune system to various autoantigens [27]. Alluded to genetic factors, genetic polymorphisms of the FcɣRIIB which are prevalent in African and Asian populations corresponding to the areas where malaria is endemic, are SLE-predisposing factors in these populations as a result of impaired handling of apoptotic body and immune complexes [28,29]. With reference to the discussion of affinity B cell selection in the germinal centers in Section 1.3, a recent study advocated the pathogenic potential of a lupus-susceptible hypofunctional polymorphism (Ile232Thr) of *FcγRIIb* in reducing apoptosis of germinal center light-zone B cells and affinity maturation without affecting the follicular helper T cells in the FcγRIIb^232Thr/Thr^ mouse model [30]. Aside from mediating apoptosis, FcɣRIIB thus potentially exerts an impact on B cell and hence the pathogenesis of SLE.

At the time of writing of this manuscript, the impact of the thymic central tolerance mechanism on the pathogenesis of SLE is largely unknown. While there are several case reports describing resolution of SLE following thymectomy [31,32,33,34], further investigation is required to answer how the thymic T cell selection processes are altered in SLE.

### 1.5. Contribution of Dysregulated Innate Immune System and Type-1 Interferon to SLE

Activation of the innate immune system is crucial to the pathogenesis of SLE. Upon sensing nuclear autoantigens complexed with their corresponding autoantibodies by the intracellular Toll-like receptors (TLR) 7 and 9 in the plasmacytoid dendritic cells (pDC), a substantially strong expression of type 1 interferon, particularly IFNα, is triggered [35,36]. While type I interferon is conventionally known to be an important effector cytokine produced by the pDC and NK cells upon sensing virus-derived nucleic acids which suppresses viral replication [37], accumulating evidence supports the strong pathogenic role of augmented IFNα signature and signaling in driving lupus disease activity and more severe clinical phenotypes of SLE [38]. Gene expression analyses have demonstrated up-regulation of interferon-inducible genes (IFNIG) in more than 50% of patients with SLE [39,40,41]. Besides the fact that IFNα activity differs between SLE patients from different ancestral backgrounds [42], gene expression and activation of interferon-related pathways appear to be related to the presence of certain autoantibodies. For instance, activation of interferon activity depends on the presence of anti-RBP antibodies in African-American SLE patients, but not among European SLE patients, whose anti-RBP and anti-dsDNA negativity was shown to drive IFNIG [43]. Recently, a transcriptome profiling study of SLE patients identified that the expression of a long-non-coding RNA (lncRNA) sequence (lnc00513), which is a strong regulator of IFNα expression, was enhanced by the SLE-risk alleles of rs205764 and rs547311. Increased expression of lnc00513 correlates with interferon signature and higher SLE disease activity [44]. While these studies on IFNIG highlight the substantial involvement of genetic polymorphism in SLE susceptibility and resultant differences of clinical manifestations in SLE patients of different ancestral backgrounds, it offers a unique opportunity to materialize more personalized treatment in such a clinically heterogeneous disease. For instance, lupus patients with high interferon signature were shown to respond superiorly to humanized anti-IFNα therapy compared to patients without interferon signature in two phase II clinical trials evaluating the safety and efficacy of anti-IFNα therapies [45].

## 2. Genetics in SLE

### 2.1. A Brief Background

The genetic contribution to the development of SLE is considerably high, which is estimated to be 66% of heritability in twin studies [46]. Over the past decade, genome-wide association studies (GWAS) have greatly improved our understanding of the genetic basis of SLE [47]. GWAS, a hypothesis-free approach, deploys genome-wide single nucleotide polymorphism (SNP) arrays to genotype 500,000 to 5,000,000 SNPs in patients and healthy individuals. The typed variants generally cover most of the untyped common variants in the human genome based on the linkage disequilibrium (LD) between typed and untyped variants [48]. High-density SNP analysis has identified and facilitated to focus on disease-associated loci where patients and healthy controls exhibit different frequencies of trait-associated alleles which are potential disease-causal variants or their proxies [47]. To date, about 100 SLE susceptibility loci have been identified, mostly in European and Asian populations [49,50,51,52,53,54,55,56,57,58,59,60,61,62,63,64,65,66,67,68,69,70,71,72,73,74,75,76,77,78,79,80,81,82,83,84,85,86], explaining the heritability of SLE up to around 30% [74,75].

### 2.2. Classical and Non-Classical Human Leukocyte Antigen (HLA) Locus in SLE

Of the SLE loci, the major histocompatibility complex (MHC) region of chromosome band 6p21 is known to be most polymorphic in the human genome [87], consisting of MHC class I (encoding HLA-A, -B, -C, -E, -F and -G), class II (encoding HLA-DP, -DM, -DO, -DQ and -DR) and class III genes (encoding complement system and inflammatory genes) [88]. High degree of genetic diversity and linkage disequilibrium within and around HLA genes has posed considerable challenges in typing HLA classical alleles and targeting causal genes and variants in large populations due to the high cost and low throughput methods for genotyping. On the other hand, HLA imputation allows accurate inference of individual-level HLA classical alleles at two-field resolution (indicating primary protein sequences for the alleles) from neighboring SNPs based on ethnicity-matched reference information [89,90,91]. HLA imputation has been applied to large-scale case-control genetic studies to investigate SNP and HLA classical alleles simultaneously and pinpoint causal variants in various autoimmune diseases including SLE.

**Figure 1:**
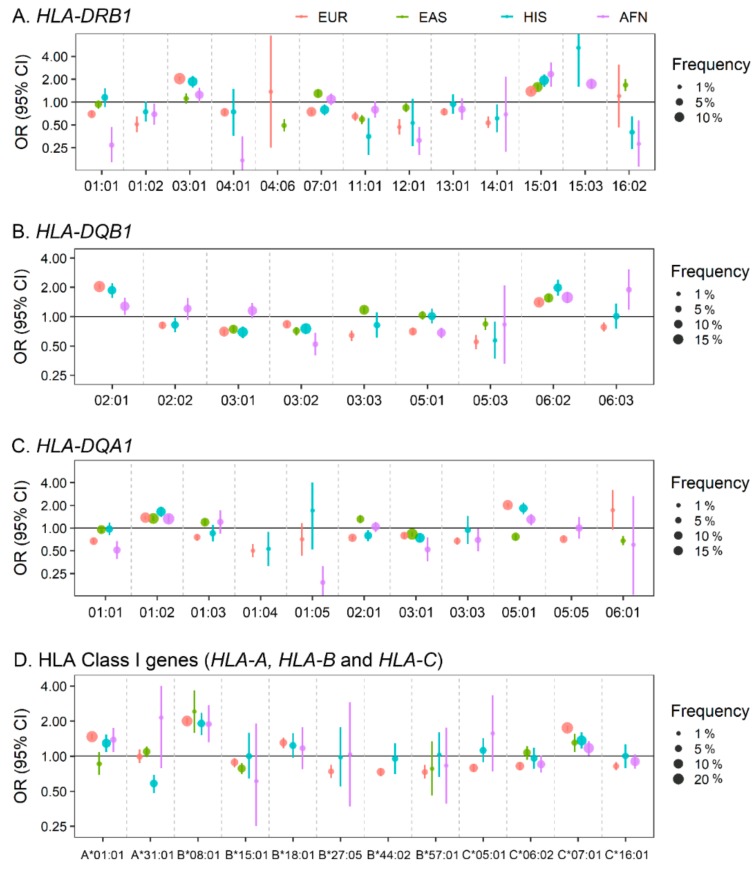
Forest plot of associations of human leukocyte antigen (HLA) classical alleles with susceptibility to systemic lupus erythematosus (SLE) in four populations. A total of 45 HLA two-field classical alleles in 6 HLA genes including (**A**) *HLA-DRB1* (13 alleles), (**B**) *HLA-DQB1* (9 alleles), (**C**) *HLA-DQA1* (11 alleles) and (**D**) HLA class I genes (2 *HLA-A* alleles, 6 *HLA-B* alleles and 4 *HLA-C* alleles) have been associated with risk of SLE at the association significance threshold p < 5.0 × 10^–6^ in at least one of four populations including European, East Asian, Hispanic and African ancestry [79,95]. The circles and error bars represent odds ratios and 95% confidence intervals, respectively. Circle sizes are proportional to allele frequencies in patients with SLE in European (orange), East Asian (green), Hispanic (blue), and African-American (purple) population. EUR: European ancestry; EAS: East Asian ancestry; HIS: Hispanic ancestry; AFN: African-American ancestry.

The primary SLE association signal in the entire MHC region has long been identified to be located at *HLA-DRB1* in the MHC class II regions or *HLA-DRB1*-associated long-range HLA-gene haplotypes in multiple ancestral populations (Figure 1). Nevertheless, it has been difficult to identify which genetic variants drive the development of SLE since ethnicity-specific linkage disequilibria and allelic heterogeneity lead to highly inconsistent allelic associations among populations. For example, with regard to the *HLA-DRB1* classical alleles, Asian SLE patients were characterized by possession of more copies of HLA-DRB1*09:01 and HLA-DRB1*15:01 (and its correlated HLA-DQB1*06:02) than healthy individuals [75,77,92]. On the other hand, in European SLE studies, the excess risk of SLE was apparently conferred by HLA-DRB1*03:01, HLA-DRB1*08:01, HLA-DQA1*01:02 and two MHC SNPs [93] or HLA-B*08:01, HLA-B*18:01, HLADQB1*02:01, HLA-DRB3*02:00 and HLA-DQA*01:02 and the class III SNP rs74290525 [51]. A recent trans-ancestral study demonstrated that HLA-DRB1*03:01-HLA-DQA1*05:01-HLA-DQB1*02:01 and DRB1*15:01/03-DQA1*01:02-DQB1*06:01 were SLE-risk haplotypes in MHC class II alleles which are shared across ancestries including European, African and Hispanic Amerindian ancestries [79]. 

Highly ethnicity-specific HLA associations in the level of HLA classical alleles have been alternatively explained by a refined analysis of HLA amino acid residues, being less influenced by the ethnicity-specific classical allele frequencies. HLA imputation and HLA-DRB1 amino acid-level analysis by Kim et al. [94] in 2013 hitherto identified the genomic elements that encode HLA-DRB1 amino acid positions 11, 13 and 26 at the epitope-biding groove of HLA-DR molecules which were associated with SLE. A recent follow-up study by Molineros et al. suggested that HLA amino acid changes in several major HLA genes were the sources of primary SLE association signals in a large population by further localizing SLE-MHC association signals at specific HLA amino-acid residues [95]. Specifically, amino-acid positions 11 and 13 in HLA-DRB1 were most significantly associated with susceptibility to SLE. A step-wise conditional analysis further localized the association signals to HLA-DRB1 amino acid position 37 (of which the association significance level was comparable with that of position 26), HLA-A position 70, HLA-DPB1 position 35, HLA-DQB1 position 37 and HLA-B position 9 [95]. Although the HLA molecules have tertiary and quaternary structures organized by the high-order interactions among multiple amino acids, primary protein sequence analysis revealed relatively consistent association among ancestries [94], providing a hint as to why association heterogeneity among ancestries appears. 

Since HLA molecules play a crucial role in autoantibody production, it is tempting to explore whether SLE-associated HLA variants contribute to autoantibody production and lupus-mediated tissue damage. Indeed, in the amino acid level, the lead SLE-associated positions 11 and 13 are not only associated with SLE susceptibility but also autoantibody production which drives lupus nephritis [94,95]. It is also noted that certain amino acid residues in the peptide-binding groove of both HLA class I and II loci are characterized by predominantly negatively charged side-chain, which correlates with overall SLE risk and with autoantibody production [95]. A Japanese SLE study identified a correlation between HLA-DRB1*04:05 and positivity of antinuclear antibodies [96]. In the Chinese population, an HLA association analysis of patients with lupus nephritis and healthy individuals identified five independent risk variants at HLA-DRB1 amino acid position 11, HLA-DQB1 amino acid position 45, HLA-A amino acid position 156, HLA-DPB1 amino acid position 76 and a missense variant in PRRC2A [97]. 

Non-classical HLA genes, MHC III genes and MHC SNPs have also been reported to be associated with SLE. MHC association fine-mapping and candidate-gene studies suggested independent contributions of several MHC SNPs outside of HLA genes [51,79,93] and an eQTL variant rs371194629 with 14-bp insertion/deletion in the 3’ untranslated region of *HLA-G* to SLE [98]. Interestingly, one of the strongest single genetic factors for SLE was observed in patients with homozygous deficiency of the *C4A* gene (OR = 12), although such deficiency is exceedingly rare [99]. The deleted *C4A* alleles are linked with the well-known SLE-risk alleles HLA-DRB1*03:01 and HLA-DRB1*15:01 in European and Asian populations, respectively. Based on this observation, the independent contribution of *HLA-DRB1* and *C4A* variants to SLE requires further validation. 

### 2.3. Non-HLA Loci in SLE

The highly polygenic etiology of SLE is supported by a large number of disease-associated loci that have modest effect sizes but surpass the genome-wide significance threshold for the genetic association with SLE [49,50,51,52,53,54,55,56,57,58,59,60,61,62,63,64,65,66,67,68,69,70,71,72,73,74,75,76,77,78,79,80,81,82,83,84,85,86]. The proportion of phenotypic variances that is explained by HLA and non-HLA variants has been estimated to be 2.6% [95] and ~28% [74,75], respectively. As sample sizes of genetic studies have been increasing, statistical power to detect small-effect SLE variants has been augmented, allowing the discovery of more potential loci that are associated with the risk of development of SLE. Furthermore, trans-ancestral analysis with high-coverage and ethnicity-specific SNP arrays has facilitated the identification of novel SLE loci and improved localization of causal variants with sound statistical and bioinformatics methods. 

**Figure 2:**
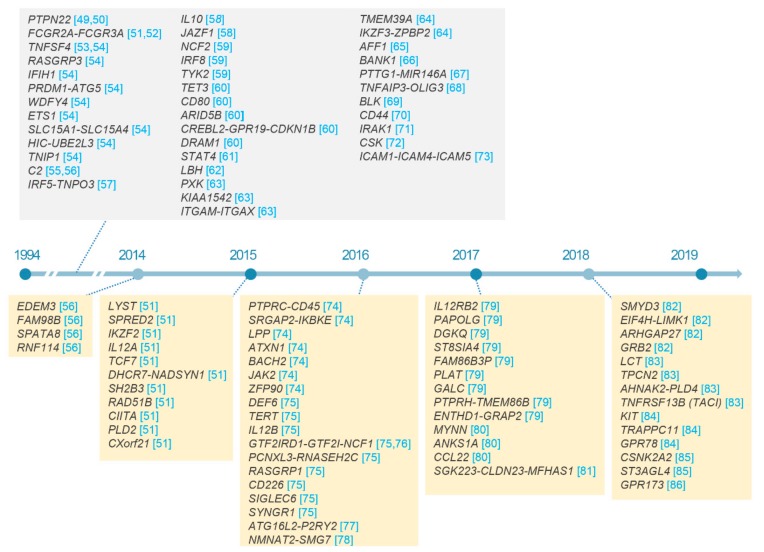
SLE susceptibility loci discovered over the past five years. Non-HLA loci associated with SLE susceptibility with p < 5.0 × 10^−8^ in previous studies, especially from 2014 to 2019, are listed with citation of references.

At the time of writing this review, there have been about a hundred non-HLA loci identified which are associated with SLE susceptibility (Figure 2) [49,50,51,52,53,54,55,56,57,58,59,60,61,62,63,64,65,66,67,68,69,70,71,72,73,74,75,76,77,78,79,80,81,82,83,84,85,86]. Most association signals are explained by common variants with modest effect sizes and pleiotropic effects on risk of other inflammatory disorders [47]. For example, the largest-ever effect size in non-HLA loci has recently been detected as a missense variant (g.74779296G > A; p.Arg90His; odds ratios of 2.02 to 3.82; risk allele frequencies of 2.1 to 18.1% in controls) in *NCF1* in multi-ethnic populations using Immunochip array and locus-targeted resequencing [76]. The p.Arg90His substitution in *NCF1* encoding the p47phox subunit of the phagocyte NADPH oxidase was reported to cause the reduction of reactive oxygen species [100,101] in patients with SLE and other autoimmune conditions including primary Sjögren’s syndrome and rheumatoid arthritis (RA) [76]. Furthermore, reduced copy number of *NCF1* has recently been found to be independently associated with the susceptibility of SLE [76]. The reason that the extremely large-effect common variants in *NCF1* have not been detected is because there have been no SNP array covering the genetically complex *NCF1* locus. 

International effort in studying SLE genetics which involves various ancestral populations has not only revealed statistically reliable SLE loci which explain a fraction of SLE heritability but also the heterogeneity of SLE variants among ancestries. One of the largest cross-ethnicity genome-wide meta-analyses in the Chinese and European populations revealed that the human genome of the two populations were substantially shared in terms of the significance and effect estimates of SLE association [74]. Most association signals displayed in Manhattan plots for each of the two populations were highly consistent between each other, especially in the reported SLE-risk loci, showing no evidence of association heterogeneity in Cochran’s Q tests but a significant correlation of the strength of association. Some population-specific differences in SLE-susceptibility associations were explained simply by the differences in allele frequencies of Bayesian-credibility SNP sets [74]. In contrast to the high degree of risk-allele sharing between the Chinese and European populations, meta-analysis using Immunochip variants in the European, African and Hispanic ancestries highlighted the presence of population-specific SNP associations across the human genome by population admixture and association analysis of SLE risk alleles [79].

The frequencies of SLE risk alleles in the general population help to evaluate each individual genetic susceptibility by means of cumulative weighted genetic risk score (wGRS), which is defined as the sum of the number of risk alleles at various loci in an individual weighted by the natural logarithm of their odds ratios [102]. The frequencies of SLE risk alleles in a general population determine the genetic susceptibility of a particular population. A wGRS analysis of five general populations found that high-wGRS populations carrying high frequencies of SLE risk alleles have higher prevalence of SLE [74]. The population order from the lowest to highest average of wGRS was as follows: Europeans < Amerindian ≈ South Asians < East Asian < Africans [74]. In addition, high wGRS for SLE was found to be more frequent in patients with childhood-onset SLE, anti-dsDNA positivity, oral ulcers and immunologic renal as well as hematologic manifestations [102,103,104], suggesting that that the high genetic load on SLE risk is not only associated with a high degree of susceptibility to SLE but also early onset and unfavorable prognosis of SLE. 

## 3. Immunobiological Insights from SLE-Risk Variants

Most non-HLA loci which are associated with SLE were mapped at non-coding variants without protein-altering proxy variants, strongly implying that the non-coding SLE-associated variants may play a regulatory role in the expression of neighboring genes and an allele-specific expression of the SLE-relevant gene could be a primary molecular phenotype in the development of SLE. As the regulatory regions featured by epigenetic annotations are highly cell-type specific, it is expected that most SLE-relevant cell types are enriched with SLE-risk variants within their regulatory genomic regions and they express more SLE-loci genes than other irrelevant cell types. Based on this hypothesis, several cell types have been implicated as key immune cell types which shape the immune system in the setting of SLE [51,75,80,105,106,107,108]. 

Epstein‒Barr virus (EBV)-infected B cells have been identified to be involved in the pathogenesis of SLE. Harley et al. [105] discovered the significant overlap between SLE-risk loci and experimentally validated binding sites of EBV-encoded EBNA2 proteins. Of the 53 SLE GWAS loci which were identified in European population, less than 26 were bound by EBNA2 and other co-occupying transcription factors, especially NF-κB proteins such as RELA, RELB, REL, NFKB1 and NFKB2, forming the so-called super-enhancers [105]. Gene expression and Chromatin ImmunoPrecipitation followed by sequencing (ChIP-Seq) analysis illustrated that the binding affinity of EBNA2-associated protein complexes to the SLE-risk loci can be altered by the SLE variants, which eventually lead to the regulation of nearby genes such as *IKZF2*, *CLEC16A*, *BLK*, *MIR3142* and *HLA-DQB1* in an allele-specific manner [105]. 

The potential contribution of the genetics aspect by various leucocyte subsets to SLE has been described. For example, the expression of the SLE-loci genes and their murine homologous genes were specific to various B-cell types [75,80]. In addition, SLE variants (and their proxy SNPs) were significantly enriched in a highly cell type-specific histone mark and tri-methylation of histone H3 at lysine 4 (H3K4me3) in B cells, T cells and monocytes [106,107,108]. Similarly, Hui-Yuen et al. found that a large fraction of the SLE-risk loci lies within or near the enhancer-associated histone marks in adult neutrophils, CD4^+^ T cells and B cells. In contrast, Bentham et al. [51] emphasized that genes encoding transcription factors in SLE loci (16 genes out of 43 likely causal genes) are significantly more frequent than expected (9 genes) and expressed broadly across various cell types. The allele-specific regulation of those transcription factors can extensively alter the regulatory expression networks in various immune cell types in the innate and adaptive immune systems [51]. 

Pathway enrichment analysis is a statistical approach to identify disease-relevant pathways where disease-loci genes are significantly enriched, although such analysis highly relies on the manually curated gene lists of biological processes and the methods that calculate enrichment scores for each biological process [109]. A pathway scoring analysis in the meta-analysis of GWAS in the Spanish and European populations identified the significant enrichment of SLE-associated genes in the pathways involved in B-cell receptor signaling, CTLA4 co-stimulation during T cell activation, interleukin-4 signaling and cell surface interactions at the vascular wall [82]. A gene set enrichment analysis using SLE-loci genes in Asians and predefined gene ontology terms identified the signal pathways related to lymphocyte activation, regulation of immune response and proliferation and activation of immune cells [75]. 

## 4. Future Directions in Genetics of SLE and Conclusion

### 4.1. Integrative Analysis of Multi-Omics Data

The understanding of SLE-risk variants in individuals can be extended to espouse variant-driven intermediate alterations at various levels, including epigenetics, transcriptomics, proteomics and immunophenotype. Integrative analysis between SLE genetics and other omics along with well-organized clinical data is increasingly recognized as an important platform to enhance the understanding of the biological and clinical heterogeneity of SLE.

Tremendous efforts to capture molecular features from the inflamed tissues in SLE patients have accumulated invaluable non-genetic data that can be analyzed in concert with the information of individuals’ SLE-risk variants in order to further understand the pathogenesis of SLE and facilitate clinical classification and prognostication of the condition. For example, a recent longitudinal blood transcriptomics study in 158 patients with pediatric SLE which attempted to address the molecular heterogeneity of SLE identified specific transcriptomic and immunological module signatures associated with clinical disease activity, active nephritis and response to treatment [110]. The patient groups, stratified based on transcriptional module signatures associated with disease activity, were genetically distinguished from one another by a large number of group-specific cis-regulatory variants [110].

To better dissect and control for the intrinsic cell-to-cell heterogeneity of gene expression in the most disease-relevant tissues and organs such as blood, kidneys and skin, a single-cell omics technology has emerged to offer innovative solutions for looking at transcriptomic and epigenetic landscapes at a single cell resolution [111,112]. Recently, the Accelerating Medicines Partnership (AMP) RA/SLE consortium performed transcriptomic studies which uncovered complex cell clusters of peripheral, kidney and urine leukocytes [113] as well as non-immune cells in the kidneys and skin [114] from patients with lupus nephritis and compared with those of healthy controls. Gene expression in renal leukocytes and renal tubular cells was respectively correlated with that in urine leukocytes and keratinocytes, demonstrating its potential as an alternative to invasive procedures to diagnose and characterize renal lupus. These studies demonstrated that strong interferon-response and fibrotic gene signatures in renal tubular cells and skin keratinocytes were associated with failure to respond to treatment for lupus nephritis [114]. Biological pathways enriched for the genes that were differentially expressed among classes of lupus nephritis supported the histopathological classification of lupus nephritis [114]. Expression analysis of renal immune cells revealed that a large number of SLE-loci genes are expressed highly specific to immune-cell clusters [113], which might provide an insight into how SLE-risk variants contribute to lupus nephritis by linking the expression of SLE-risk genes in relevant renal cell populations.

Single-cell transcriptomics technology has recently been applied in SLE research for the deconstruction of molecular and cellular heterogeneity among patients with the condition. In addition, single-cell epigenomics will likely become an important method to understand the molecular mechanisms about the cell type-specific regulation of immune-related genes in a disease condition. Furthermore, integrative analysis to simultaneously recapitulate disease alleles and cell-to-cell variations in the immune system using genetic and single-cell omics data will fill the gap between cell type-specific molecular alterations and known SLE-risk loci, providing a new opportunity to identify novel and clinically relevant diagnostic and prognostic biomarkers.

### 4.2. Conclusions

Through a powerful hypothesis-free approach to scanning susceptibility loci of common complex traits with GWAS, around 100 lupus-susceptibility loci have been identified in large scale cohorts, which further improves our understanding of the genetic architecture of SLE. A large portion of SLE heritability, however, is suspected to be hidden behind a large number of common genetic variants with small effect sizes and a few rare variants with large effect sizes [115].

The potential clinical application of SLE GWAS for precision medicine and therapeutic target discovery remains challenging. Nevertheless, GWAS has been found to be useful in prioritizing causal genes in SLE loci with other biological information and locating some approved SLE drugs and repurposable drugs in the protein-protein networks derived from SLE-susceptibility genes [75]. Similarly, a recently developed framework for the prioritization of drug targets based on functional genomic features, immune-related annotations and network connectivity in RA showed that top prioritized genes were linked to a few approved drugs such as abatacept, tocilizumab, and TNF-inhibitors [116,117,118]. Drugs that target disease-loci genes are twice as likely to be approved from phase 1 clinical trials [119], demonstrating the potential clinical applicability of SLE genetics in enhancing drug development and validating therapeutic targets.

Through integrative analysis involving existing and newly emerging multi-omics data, the discovery of more SLE-risk variants will provide us important clues as to the SLE-relevant cell types and signaling pathways involved in the pathogenesis of SLE. Such knowledge will optimistically lead to the expansion of the armamentarium of diagnostic and prognostic biomarkers of SLE, as well as druggable targets in the realm of genetics which can be further tested.
